# Development and implementation of ‘A guide to PPIE – Early Integration into Research Proposals’ in a multi-disciplinary consortium

**DOI:** 10.1093/rheumatology/kead482

**Published:** 2023-09-14

**Authors:** Richard Beesley, Freya Luling Feilding, CLUSTER Consortium Champions, Richard Beesley, Sharon Douglas, Emily Earle, Nick Gannon, Kerry Leslie, Eilean MacDonald, Debbie Wilson, Catherine Wright, Elizabeth C Rosser, Stephanie J W Shoop-Worrall, Alyssia McNeece, Zoe Wanstall, Kimme Hyrich, Lucy R Wedderburn, the CLUSTER Consortium, Lucy R Wedderburn, Melissa Kartawinata, Zoe Wanstall, Bethany R Jebson, Freya Luling Feilding, Alyssia McNeece, Elizabeth Ralph, Vasiliki Alexiou, Fatjon Dekaj, Aline Kimonyo, Fatema Merali, Emma Sumner, Emily Robinson, Andrew Dick, Michael W Beresford, Emil Carlsson, Joanna Fairlie, Jenna F Gritzfeld, Athimalaipet Ramanan, Teresa Duerr, Michael Barnes, Sandra Ng, Kimme Hyrich, Stephen Eyre, Soumya Raychaudhuri, Andrew Morris, Annie Yarwood, Samantha Smith, Stevie Shoop-Worrall, Saskia Lawson-Tovey, John Bowes, Paul Martin, Melissa Tordoff, Jeronee Jennycloss, Michael Stadler, Wendy Thomson, Damian Tarasek, Chris Wallace, Wei-Yu Lin, Nophar Geifman, Sarah Clarke, Victoria J Burton, Thierry Sornasse, Daniela Dastros-Pitei, Sumanta Mukherjee, Michael McLean, Anna Barkaway, Victoria Basey, Peyman Adjamian, Helen Neale, John Ioannou, Hussein Al-Mossawi

**Affiliations:** Juvenile Arthritis Research, Tonbridge, UK; Infection Immunity and Inflammation Research and Teaching Department UCL Great Ormond Street Institute of Child Health, London, UK; Centre for Adolescent Rheumatology Versus Arthritis at UCL, University College London Hospitals (UCLH) and Great Ormond Street Hospital (GOSH), London, UK; Centre for Rheumatology Research, Division of Medicine, University College London, London, UK; Centre for Epidemiology, Faculty of Biology, Medicine and Health, The University of Manchester, Manchester Academic Health Sciences Centre, Manchester, UK; Infection Immunity and Inflammation Research and Teaching Department UCL Great Ormond Street Institute of Child Health, London, UK; Infection Immunity and Inflammation Research and Teaching Department UCL Great Ormond Street Institute of Child Health, London, UK; Centre for Epidemiology, Faculty of Biology, Medicine and Health, The University of Manchester, Manchester Academic Health Sciences Centre, Manchester, UK; National Institute of Health Research Manchester Biomedical Research Centre, Manchester University NHS Trust, Manchester, UK; Infection Immunity and Inflammation Research and Teaching Department UCL Great Ormond Street Institute of Child Health, London, UK; Centre for Adolescent Rheumatology Versus Arthritis at UCL, University College London Hospitals (UCLH) and Great Ormond Street Hospital (GOSH), London, UK; National Institute of Health Research Biomedical Research Centre at GOSH, London, UK

Rheumatology key message

- Systematic early involvement of patients and patient representatives in research planning enables patient-centred research outputs.

Dear Editor, Patient and Public Involvement and Engagement (PPIE) is a critical part of research, from early study design through to dissemination of results. The importance of PPIE input in the early phases of research design is significant and increasingly recognized throughout the research community, with most funders now requiring evidence of meaningful PPIE in grant applications [1]. Benefits include more patient-oriented research goals and creating a communication network for dissemination of study findings to the public. Thus, PPIE forms a fundamental part of delivering effective, impactful research.

Several approaches have been developed to involve patients closely in research [2, 3]. Previous publications have proposed key factors to consider when developing patient involvement plans [4]; however, mechanisms for how best to achieve this are less clearly defined. There remains a need for step-by-step practical guidance for researchers to follow when working with patients and lay partners to reduce an imbalance of understanding, to recognize the value of non-scientific participants, and to ensure the voices and views of patients are represented. Here we describe the approach taken within the CLUSTER Consortium.

CLUSTER is a UK-wide multi-disciplinary consortium focused on precision medicine research for JIA [5]. It brings together researchers in JIA and associated JIA-uveitis, with bioinformaticians and industry partners, in partnership with a patient and parent network.

From its inception, CLUSTER developed a dedicated UK-wide PPIE group, the CLUSTER Consortium Champions, hereafter referred to as ‘The Champions’. These individuals have lived experience of JIA and/or JIA-uveitis, typically being patients or parents [6]. Several of the Champions also represent JIA charities thereby forming a wider patient and parent network, helping to diversify patient views and experiences that they bring to CLUSTER. The Champions worked closely with the Consortium’s research partners to develop an innovative PPIE policy (Fig. 1). This sets out the process to support involvement at the earliest possible stage of project design and grant applications, whilst acknowledging the real-world context of time pressures. This process integrates feedback forms (Supplementary Data S1, available at *Rheumatology* online) for researchers and PPIE participants to complete, facilitating a feedback loop to both capture the value of patient involvement (impact of implementation) and inform ongoing improvements. The CLUSTER Consortium has internal funding calls which were used to test and refine this policy. Through implementation, all researchers applying for internal funding were required to involve the Champions early in discussions, prior to submitting formal funding applications. For specific projects, typically a subset of the group take part, depending on their availability and the specific skills and interests that different individuals bring.

**Figure 1.**
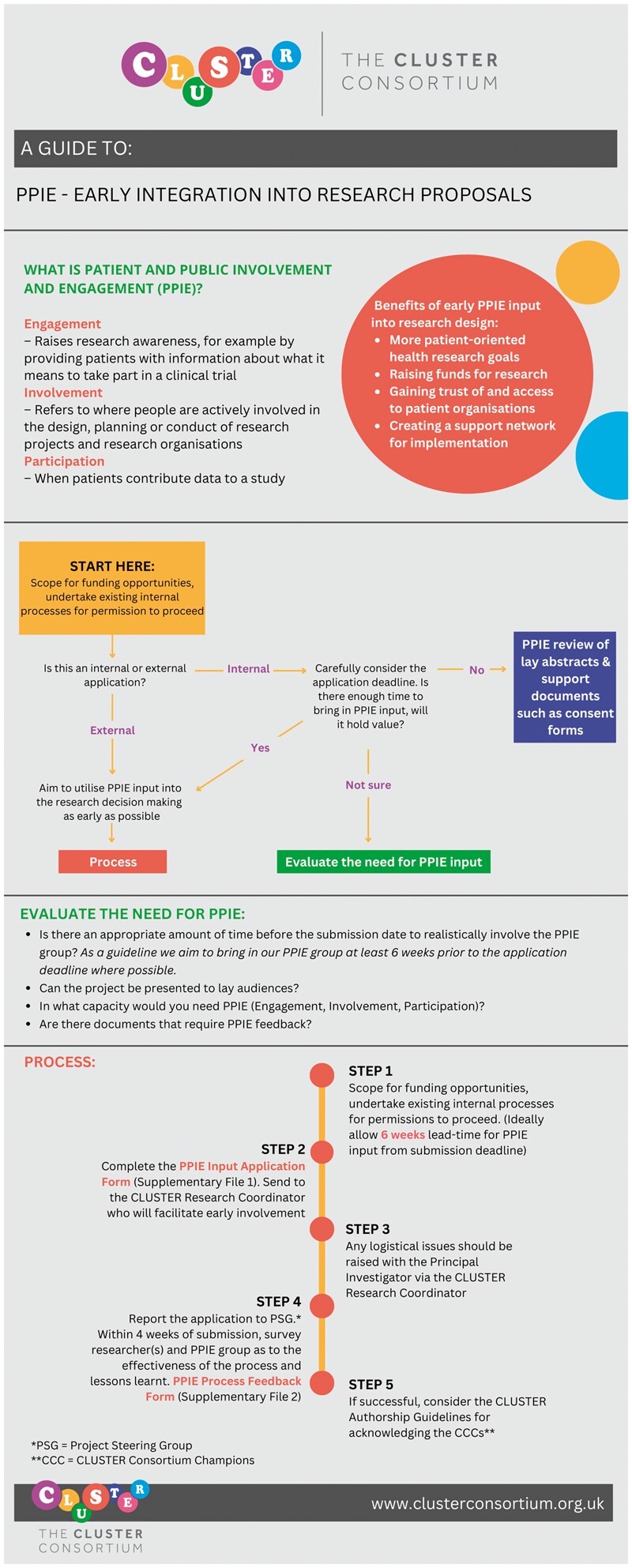
A Guide to PPIE – Early Integration into Research Proposals. Infographic summarizes the CLUSTER Consortium’s PPIE Policy for internal and external funding applications. This policy provides a stepwise process for researchers to follow when scoping for funding opportunities to facilitate involvement of patient representatives early in research planning

The positive impact of implementation is evidenced by survey data collated following each application cycle. In 80% of cases, the Champions involved scored the process 10/10 when asked how satisfied they were with their involvement. Similarly, in all cases researchers reported extremely positive experiences and cited significant changes to their proposal as a direct result of consultation with the Champions.

Two example cases demonstrate the impact of involvement facilitated by this policy. The Champions contributed to complementary projects titled ‘*Gut-derived metabolites and modulation of pathogenic B-cells in Juvenile Idiopathic Arthritis’* and ‘*Immunomodulation of pathogenic B cell responses by gut-derived metabolites in Juvenile Idiopathic Arthritis*’, which were subsequently funded as part of highly prestigious awards from the Kennedy Trust for Rheumatology Research and Foundation for Research in Rheumatology (FOREUM). Their input had a significant impact on project design, notably on the patient facing aspects including qualifying the patient impact and methods to increase the accuracy of the dietary assessment. Most significantly, this led to the Champions being included as co-applicants on the FOREUM proposal, with ongoing involvement in experimental design.

Early involvement in a second project entitled ‘*Unlock PsA: Stratifying the Impact of Psoriatic Arthritis in Children and Adults’* was also critical. The primary research question was co-developed with PPIE, identifying the right treatment from the outset of disease. The group then co-designed the project in greater depth, including which experiences should be studied, identifying a specific drug (methotrexate) as a key focus, and highlighting the importance of comparing disease in childhood and adulthood. This proposal led to a prestigious Fellowship from the Medical Research Council. With a defined process, this policy supports Early Career Researchers, many of whom have no patient-facing experience, in successful patient involvement and embeds good practice in the future leaders of Paediatric Rheumatology research.

*‘The CLUSTER champions were a fundamental part of the project development. They provided critical insight into the processes that support patient recruitment, resulting in new collaborations to enhance the collection of dietary information. This is fundamental for the accuracy of measuring gut-derived metabolites and has been transformative for the research programme’.* (Researcher 1)

‘*As a non-clinical researcher, my perception of a ‘gap’ in the research that I could fill may not lead to a meaningful research question or output. Involving the Champions has both ensured that the projects I propose are meaningful to those it is designed for and has allowed me to give agency to young people and their families in planning research that matters to them’.* (Researcher 2)

Allowing adequate time for meaningful involvement was a key area of improvement echoed in both cases. The latest iteration of the policy mandates a 6-week lead time to involve the Champions in grant proposals where possible. Consultation with both groups also gave rise to the ‘PPIE Application Form’ (Supplementary Data S2, available at *Rheumatology* online). Completion of this form by researchers when requesting Champion involvement helps to set clear expectations for both parties and increases efficiency.

The importance of involving patients and parents or carers early in the research process cannot be overstated. This makes it more likely that relevant research questions are asked and that the intended outcomes respond to patients’ unmet needs, informed by lived experience. This clearly defined strategic policy has enabled systematic incorporation of PPIE into the early phases of research planning in CLUSTER and high-quality patient involvement has been demonstrated throughout the project. Ultimately this approach will strengthen research outcomes, maximising benefit for patients with JIA and JIA-Uveitis.

## Supplementary Material

kead482_Supplementary_Data

## Data Availability

The data underlying this article will be shared on reasonable request to the corresponding author.

## References

[1] Looking forward: Working with the Medical Research Council towards a public involvement strategy main report. 2022. https://www.ukri.org/wp-content/uploads/2023/02/MRC-10023-PublicInvolvementReview2022-MainReport.pdf (31 March 2023, date last accessed).

[2] Kaisler RE , Missbach B. Co-creating a patient and public involvement and engagement ‘how to’ guide for researchers. Res Involv Engagem 2020;6:32. 32566249 10.1186/s40900-020-00208-3 PMC7301967

[3] Smits DW , van Meeteren K, Klem M, Alsem M, Ketelaar M. Designing a tool to support patient and public involvement in research projects: the Involvement Matrix. Res Involv Engagem 2020;6:30. 32550002 10.1186/s40900-020-00188-4 PMC7296703

[4] Hoddinott P , Pollock A, O'Cathain A et al How to incorporate patient and public perspectives into the design and conduct of research. F1000Res 2018;7:752. 30364075 10.12688/f1000research.15162.1 PMC6192439

[5] The CLUSTER Consortium. https://www.clusterconsortium.org.uk/ (25 January 2023, date last accessed).

[6] The CLUSTER Consortium. 2019. https://www.clusterconsortium.org.uk/cluster-consortium-champions/ (3 August 2023, date last accessed).

