# Supplementary material for: Development and implementation of ‘A guide to PPIE – Early Integration into Research Proposals’ in a multi-disciplinary consortium
Source: Rheumatology (Oxford). 2023 Sep 14;63(3):e88–91. doi: 10.1093/rheumatology/kead482 (PMC10907810; doi:10.1093/rheumatology/kead482)
Supplement: kead482_Supplementary_Data [file kead482_supplementary_data.zip › kead482_Supplementary_Data/rhe-23-1257-File001.docx]

# **CLUSTER PPIE: Early Integration into Research Proposals**

# **PPIE Process Feedback Form**

To ensure the effectiveness of early PPIE involvement in all bid-applications, we request that feedback is collected from both researcher(s) and the CLUSTER Consortium Champions within **4 weeks** of application submission. These will be distributed by the CLUSTER Research Coordinator and the completed forms will be reviewed and discussed at Project Steering Group Meeting (PSG).

| **Application Reference Number (admin use only)** |  |
| --- | --- |

| PPIE Process Feedback Form – for completion by the RESEARCHER | |
| --- | --- |
| Investigator(s) |  |
| Applicant organisation(s) |  |
| Date of Application |  |
| Was the application successful? |  |
| How did the PPI/E input aid the design of the project? |  |
| Breakdown of tasks that the CCCs helped with |  |
| Would you approach this differently if requiring PPI/E input again? If yes, please explain why. |  |

Part 1 – Feedback document to be completed by the researcher/s after the grant application has been submitted.

| **Application Reference Number (admin use only)** |  |
| --- | --- |

| PPIE Process Feedback Form - for completion by the CHAMPIONS | |
| --- | --- |
| CLUSTER Investigator(s) |  |
| Date of Application |  |
| How satisfied are you with your involvement in this application? Please rate on a scale of 1 to 10 and explain your answer |  |
| Were you given enough notice prior to meetings being organised? |  |
| If there was any additional reading or training required from you, were you supported appropriately? If no, please explain your answer |  |
| Is there anything we can do to improve your experience for future grant applications? |  |

Part 2 – Feedback document to be completed by the CLUSTER Consortium Champions after the grant application has been submitted.
