# Supplementary material for: Development and implementation of ‘A guide to PPIE – Early Integration into Research Proposals’ in a multi-disciplinary consortium
Source: Rheumatology (Oxford). 2023 Sep 14;63(3):e88–91. doi: 10.1093/rheumatology/kead482 (PMC10907810; doi:10.1093/rheumatology/kead482)
Supplement: kead482_Supplementary_Data [file kead482_supplementary_data.zip › kead482_Supplementary_Data/rhe-23-1257-File002.docx]

**CLUSTER PPIE: Early Integration into Research Proposals**

**Application for CLUSTER Champion Involvement**

**Introduction:**

This form should be used to help you plan your initial discussions with Patient and Public Involvement and Engagement (PPIE) representatives. Please complete this form and send it to the CLUSTER Research Coordinator to distribute to the CLUSTER Champions before arranging a discussion. Your responses will help set expectations for the level of engagement and time commitment required, as well as provide necessary context and background to the project proposal.

We recognise your project plans will not be finalised at this stage, but please provide as much detail as possible. All information should use lay terms and explain any technical or specialist language where used.

**Confidential:**

| **Application Reference (admin use only)** |  |
| --- | --- |

| **Project title** |  |
| --- | --- |
| **Researcher(s) Name** |  |
| **Date of application** |  |
| **Overall Aim of Project** |  |
| **Short lay summary of project (100 words max)** |  |
| **What are the timeframes for PPIE input:**   1. **What deadlines do you have for the project?** *E.g., if you are submitting a grant application, what is the deadline?* 2. **What is the expected duration of the grant?** 3. **What is the anticipated time commitment for the patient representative?** *E.g., 'Initial 30-minute meeting in 3 weeks, and discussions each quarter throughout the project’* |  |
| **What would you like the PPIE representatives to do?** *E.g., attend a discussion, help review a grant application, take part in ongoing meetings during the project…* |  |
| **How many patient representatives are you looking to be involved and do you have any specific criteria in mind?** e.g., age, experience, skills |  |
| **What do you hope to achieve by involving PPIE representatives?** |  |
| **What do you hope to offer PPIE representatives that are involved?** (Include details of any reimbursement, training and learning opportunities, personal development) |  |
| **Please send this application by email to the CLUSTER Research Coordinator** | |
